# From community support to subjective well-being: an integrative moderated mediation model of resilience and parenting stress in rural contexts

**DOI:** 10.3389/fpsyg.2026.1926635

**Published:** 2026-08-10

**Authors:** Lu Xing, Xueqi Wu

**Affiliations:** 1 School of Preschool Education, Changsha Normal University, Changsha, China; 2 School of Education, Faculty of Social Sciences Leisure Management, Taylor’s University, Subang Jaya, Malaysia; 3 Taylor’s Culinary Institute, Faculty of Social Sciences Leisure Management, Taylor’s University, Subang Jaya, Malaysia

**Keywords:** community embedded support, family demands-resources model, parenting stress, psychological resilience, rural revitalization, subjective well-being

## Abstract

**Background:**

Amidst rural revitalization, parents in rural areas navigate the complex dual responsibilities of structural socio-economic transition and intensive family education. While subjective well-being (SWB) is a critical indicator of parental psychological health, the statistical pathways that may account for the association between external community resources and internal well-being—and the boundary conditions of this process—remained underexplored.

**Methods:**

Drawing upon the Family Demands-Resources (FD-R) model and Conservation of Resources (COR) theory, this study proposed and tested an integrative model. We specified psychological resilience as a statistical mediator of the association between community embedded support and SWB, with parenting stress moderating the latter stage of this indirect association. A cross-sectional survey was conducted among 358 rural parents (males 30.45%, females 69.55%), utilizing validated psychometric instruments (WHO-5, CD-RISC, PSI-SF, and PCSQ). Structural equation modeling (SEM) was employed to examine the structural relationships.

**Results:**

The findings indicated that CES was positively associated with SWB, with psychological resilience serving as a significant partial mediator. Furthermore, parenting stress moderated the latter stage of the indirect association. The index of moderated mediation was significant, and the conditional indirect association between CES and SWB through psychological resilience became weaker as parenting stress increased. This pattern was consistent with a resource-depletion explanation.

**Conclusion:**

These findings highlight the joint relevance of community resources, psychological resilience, and parenting stress to rural parents’ SWB. Psychological resilience may help explain the association between CES and SWB, but this indirect association was weaker among parents reporting higher parenting stress.

## 1 Introduction

In the contemporary era of rapid globalization and systemic socio-economic transitions, the ecology of family life has undergone a profound transformation worldwide. Modernization has not only reshaped economic structures but also fundamentally redefined family dynamics, leading to the global emergence of “intensive parenting” (Doepke, 2019; Hays, 1996). This pervasive normative shift places unprecedented emotional, cognitive, and financial demands on parents, tasking them with the overwhelming responsibility of ensuring their children’s educational and social success in an increasingly competitive environment. Consequently, the subjective well-being (SWB) of parents—conceptualized as individuals’ cognitive and affective evaluations of their lives (Diener et al., 1999)—has become a critical focal point in contemporary positive psychology, particularly when examining resilience across diverse contexts. Grounded in ecological systems theory (Bronfenbrenner, 1981), parental SWB functions as a foundational determinant of the family climate and the quality of children’s developmental outcomes (Prime et al., 2020). As parents worldwide navigate these compounding structural stressors, there is a pressing global need for integrative approaches to identify the external factors that can proactively protect and sustain parental psychological health.

Narrowing down from this global landscape, rural China presents a particularly compelling contextual setting for examining these dynamics. Driven by the national Rural Revitalization strategy, the social ecology of Chinese rural communities is undergoing a profound and rapid systemic transition (Liu et al., 2020). Within this evolving environment, rural parents find themselves navigating a complex “dual burden.” On the one hand, they face persistent pressures related to livelihood development and economic adaptation as traditional agrarian structures modernize (Zhou et al., 2020). On the other hand, they are increasingly confronted with the pervasive demands of “intensive parenting,” a norm that has cascaded from urban centers into rural societies and sustained widespread educational anxiety (Chen et al., 2022). However, unlike their urban counterparts, rural parents often face a stark structural mismatch: they harbor equally high educational expectations for their children but must pursue them with relatively constrained household capital and local educational resources (N. Wang, 2024). This intense dual burden—balancing the demands of socio-economic transition with intensive family education—renders their internal psychological reservoirs highly vulnerable to depletion. Consequently, rural China serves as a critical backdrop for investigating how parental well-being can be sustained against the dual currents of structural transition and educational anxiety. At the local level, the present study focuses on parents from rural communities in Fenghuang, Sangzhi, and Huayuan counties in Hunan Province, providing a specific context in which to examine these associations.

Despite the critical importance of parental psychological health amidst these structural transitions, the existing literature exhibits two notable gaps. First, while extensive research has documented the detrimental impacts of familial demands on parental distress and burnout (Mikolajczak et al., 2018; Piotrowski et al., 2023), fewer studies have adopted an integrative positive psychology approach to explore how subjective well-being can be actively sustained across challenging ecological contexts. Second, studies that do acknowledge the benefits of external social resources frequently treat the connection between these resources and internal well-being as a direct, mechanical pathway (Cohen, 2004; Holt-Lunstad, 2022). However, contemporary systemic frameworks argue that this simplistic micro-level paradigm overlooks the intricate “black box” of cognitive processing and resource translation. Specifically, there is a distinct need to understand how meso-level ecological resources—such as community embedded support (CES)—are internalized into psychological capacities, and under what specific boundary conditions this protective process might be compromised.

To address these gaps, the present study aims to systematically examine a moderated mediation framework. By theoretically synthesizing the Demands-Resources theory—adapted for the family context (Bakker et al., 2023; Demerouti et al., 2001)—and Conservation of Resources (COR) theory (Hobfoll, 1989), this research seeks to elucidate whether meso-level structural backing is directly and indirectly associated with individual well-being through psychological resilience, and whether the strength of this indirect association differs across levels of parenting stress. Ultimately, this study identifies potentially relevant targets for community-based interventions, whose effectiveness should be evaluated in longitudinal or experimental research in rural contexts.

## 2 Literature review and hypothesis development

### 2.1 Community embedded support and subjective well-being

Subjective well-being (SWB) is a comprehensive psychological construct encompassing an individual’s cognitive evaluation of life satisfaction and the presence of positive affect (Diener et al., 2018). In the context of rural transitions, sustaining parents’ SWB requires more than individual coping mechanisms; it necessitates structural resources. Community embedded support (CES) refers to the accessible social, emotional, and instrumental resources deeply integrated within an individual’s local socio-ecological environment (Hobfoll, 2001). Unlike generic social support, which is often conceptualized as a micro-level interpersonal exchange, CES operates as a meso-level structural factor. It reflects the strength of communal ties, local resource accessibility, and the collective efficacy of the rural neighborhood.

According to the Conservation of Resources (COR) theory, individuals are fundamentally motivated to acquire, retain, and protect resources to mitigate stress and enhance well-being (Hobfoll, 1989). For rural parents navigating the dual burden of economic transition and intensive parenting demands, CES serves as a critical external resource pool. When parents are embedded in a supportive community, they can readily draw upon shared community childcare networks, emotional validation from peers, and informational resources regarding educational opportunities. Within COR theory, these meso-level resources are expected to be associated with lower perceived burden and more favorable evaluations of life. Empirical evidence across various cultural contexts has consistently demonstrated that strong community integration is a robust predictor of adult SWB (Cohen, 2004; Diener et al., 2018). Building upon these foundational findings, recent research has further highlighted the relevance of structural support, social connection, and collective resources for well-being under conditions of social and economic strain (Holt-Lunstad, 2022; Walsh, 2021; Waters et al., 2022).

Despite the general consensus regarding the protective role of social resources, a critical synthesis of the literature reveals notable inconsistencies, particularly concerning the structural level at which this support operates. Several empirical studies relying on micro-level paradigms have reported mixed or even paradoxical findings (Bolger and Amarel, 2007; Rook, 2015). Specifically, informal interpersonal support can sometimes exacerbate parental distress due to emotional contagion, the pressure of reciprocal obligations, or a fundamental mismatch between the support provided and the specific stressor experienced (Nomaguchi and Milkie, 2020; Zee and Bolger, 2019). These mixed findings suggest that the benefits of interpersonal support may depend on how well the support matches parents’ needs and on the reciprocal demands attached to it.

Against this background, the present study focuses on community embedded support at the meso-level. CES reflects resources embedded in community participation, recurring social ties, and local institutions rather than support obtained from a single interpersonal relationship. Compared with support that depends on a single interpersonal relationship, CES may provide more stable access to information, participation opportunities, and institutional assistance. These characteristics provide a theoretical basis for expecting CES to be positively associated with subjective well-being. Taken together, these theoretical and empirical arguments provide the basis for expecting a positive association between community embedded support and the subjective well-being of rural parents.

### 2.2 Mediating role of psychological resilience

To understand the “black box” through which external community resources translate into subjective well-being, this study positions psychological resilience as a central mediating mechanism. Diverging from traditional deficit-based models that view resilience merely as an innate, static trait, contemporary positive psychology conceptualizes it as a dynamic, malleable capacity that enables individuals to successfully adapt to and bounce back from structural adversities (Luthar et al., 2000; Masten, 2001).

Several psychological resources may help parents respond to chronic stress, including self-efficacy, perceived control, and psychological resilience. The present study focuses on resilience because it reflects a broad capacity to adapt across changing demands and because systemic perspectives conceptualize resilience as responsive to ecological and relational resources (Walsh, 2021). Consistent with the extended Family Demands–Resources model, community resources may therefore be associated with parents’ psychological resilience, which in turn may be associated with their subjective well-being (Bakker et al., 2023).

The linkage between CES and psychological resilience can be elegantly explained through the lens of resource internalization. According to the extended Family Demands-Resources (FD-R) model, robust external ecological resources act as the scaffolding for accumulating internal personal resources (Bakker et al., 2023). When rural parents perceive high levels of structural and emotional backing from their community—such as accessible childcare advice and communal validation—they form a secure ecological base. This supportive meso-level environment fosters a “gain spiral” (Hobfoll, 2001); it may be associated with lower perceived cognitive burden and a stronger sense of security, which may in turn be related to psychological resilience. Related research has linked supportive family and community contexts with parental resilience during periods of social or ecological transition (Prime et al., 2020). In essence, external community assets may provide an ecological context associated with stronger resilience.

In turn, this accumulated psychological resilience serves as a powerful engine for sustaining well-being. Drawing upon the broaden-and-build theory within positive psychology (Tugade and Fredrickson, 2004), highly resilient parents are better equipped to cognitively reframe the daily frictions of intensive parenting. They can effectively regulate negative affect, deploy flexible coping strategies, and maintain a proactive outlook despite rural transition stressors. This dynamic is supported by contemporary positive psychology research, which highlights how robust psychological resilience enables individuals to prevent chronic psychological drain and significantly elevate life satisfaction, even when structural or environmental stressors are prevalent (Yıldırım and Arslan, 2022). Collectively, these arguments identify psychological resilience as a plausible internal mechanism through which community embedded support may be associated with the subjective well-being of rural parents.

### 2.3 Moderating role of parenting stress

While psychological resilience generally facilitates the translation of meso-level community support into subjective well-being, this process is not immune to intense contextual demands. In the context of rural China’s rapid socio-demographic transitions under compressed modernity, parenting stress emerges as a critical boundary condition. Parenting stress is defined as the aversive psychological reaction experienced when the demands of the parenting role significantly exceed an individual’s available resources (Deater-Deckard, 2008). For rural parents navigating structural inequalities and heightened intensive parenting expectations, this stress often manifests as chronic psychological pressure.

Parenting stress may vary in intensity and duration, but the present study focuses on stress arising from persistent caregiving and educational demands. For rural parents managing intensive parenting expectations with comparatively constrained household and community resources, these demands may place continuing pressure on their available psychological resources. From a COR perspective, parenting stress may therefore function as a boundary condition associated with variation in the positive relationship between psychological resilience and subjective well-being.

This moderating role can be understood within the resource-conservation logic of COR theory. When parenting demands remain high, parents may need to allocate more psychological and emotional resources to immediate caregiving difficulties, leaving fewer resources available for emotion regulation, positive appraisal, and other adaptive coping processes. Cross-cultural research on parental burnout similarly indicates that persistent imbalances between parenting demands and available resources are associated with parental exhaustion (Roskam et al., 2021).

Accordingly, the positive association between psychological resilience and subjective well-being is expected to be weaker at higher levels of parenting stress. When parenting stress is lower, parents may have greater opportunity to use the adaptive coping capacities associated with resilience. Parenting stress is therefore proposed as a moderator of the latter stage of the indirect association between community embedded support and subjective well-being through psychological resilience.

### 2.4 Research questions

The preceding review identifies unresolved questions concerning the association between community embedded support and parental subjective well-being, the internal psychological mechanism underlying this association, and the stressful boundary conditions under which this mechanism may be attenuated. Accordingly, the present study addresses the following research questions:

*RQ1*: How is community embedded support associated with the subjective well-being of rural parents?

*RQ2*: Does psychological resilience mediate the association between community embedded support and the subjective well-being of rural parents?

*RQ3*: Does parenting stress moderate the indirect association between community embedded support and subjective well-being through psychological resilience, such that the positive association between psychological resilience and subjective well-being is weaker at higher levels of parenting stress?

### 2.5 Hypotheses

Drawing on the theoretical arguments and empirical evidence synthesized above, the following hypotheses are proposed:

*H1*: Community embedded support (CES) is positively associated with the subjective well-being (SWB) of rural parents.

*H2*: Psychological resilience significantly mediates the association between community embedded support (CES) and subjective well-being (SWB). This mediation hypothesis comprises the following two constituent paths:

*H2a*: Community embedded support (CES) is positively associated with psychological resilience (PR) among rural parents.

*H2b*: Psychological resilience (PR) is positively associated with the subjective well-being (SWB) of rural parents.

*H3*: Parenting stress significantly moderates the indirect association between community embedded support (CES) and subjective well-being (SWB) through psychological resilience. Specifically, the positive association between psychological resilience and subjective well-being is weaker when parenting stress is high than when it is low.

### 2.6 The current study

Drawing on the resource internalization perspective and Conservation of Resources theory, the present study examines whether community embedded support is associated with rural parents’ subjective well-being both directly and indirectly through psychological resilience, and whether parenting stress moderates the latter stage of this indirect association.

**FIGURE 1:**
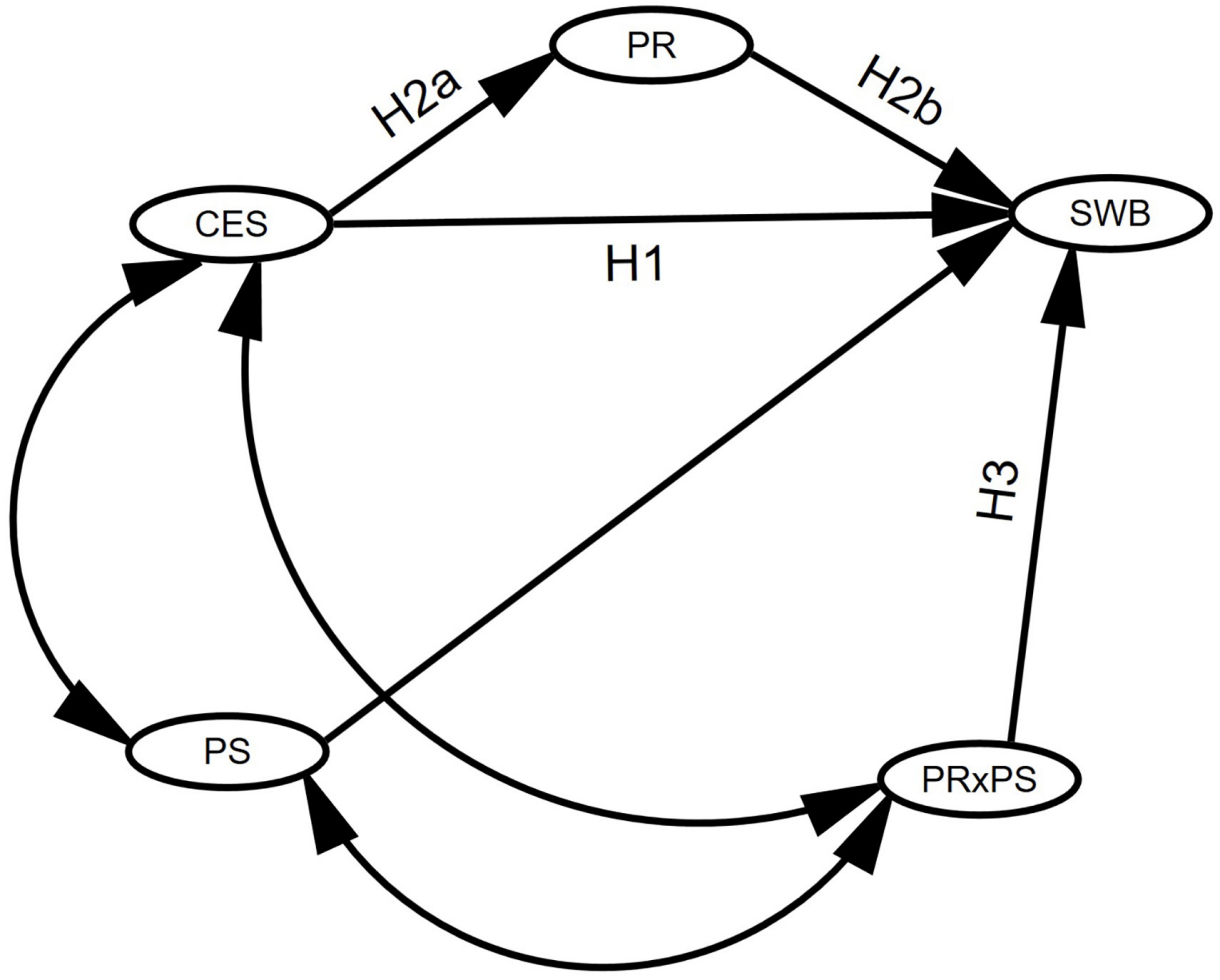
Structural model.

To test these hypotheses, a moderated mediation model was specified. The conceptual framework of the current study, which integrates the hypotheses developed in the preceding sections, is illustrated in Figure 1.

## 3 Materials and methods

### 3.1 Participants and procedure

This study employed a cross-sectional, quantitative survey design targeting rural parents within the context of China’s ongoing rural revitalization initiative. Data were collected between November 2023 and July 2024 from parents residing in 11 natural villages across Fenghuang County, Sangzhi County, and Huayuan County, Hunan Province, China. A multi-site convenience sampling approach was employed, with eligible parents recruited through village committees and rural school parent–teacher associations using both online questionnaires and in-person paper-based distribution. The inclusion of multiple villages across three counties was intended to broaden the coverage of rural community contexts, while the eligibility criteria ensured that respondents had direct experience of daily caregiving and educational supervision. Prior to participation, informed consent was obtained from all respondents. The introductory section of the survey explicitly outlined the research objectives, guaranteed absolute anonymity and confidentiality, and emphasized the voluntary nature of participation, including the right to withdraw at any stage without penalty.

To be eligible for inclusion, participants were required to meet the following operationally defined criteria: (1) hold a rural household registration (Hukou) and have continuously resided in the target rural community for at least 12 months; (2) be the primary legal guardian of at least one minor child aged between 3 and 18 years; and (3) co-reside with the target child, taking primary responsibility for their daily caregiving and educational supervision, thereby excluding absentee or heavily migrating parents to align with the study’s focus on daily parenting stress.

The initial sample size was determined through an a priori power analysis conducted in G*Power 3.1 (Faul et al., 2009), using the “Linear multiple regression: Fixed model, R^2^ deviation from zero” procedure. To ensure adequate statistical power for evaluating the structural paths, we estimated the required sample size based on a conservative small-to-medium effect size (*f*^2^ = 0.05), a standard alpha level of α = 0.05, and a desired statistical power of 1 − β = 0.95 with three main predictors. The analysis indicated a minimum requisite sample of 348 participants. Because recruitment combined paper-based questionnaire distribution with online announcements disseminated through village collective WeChat groups, the total number of eligible parents who actually received or viewed the survey invitation could not be determined. Some paper questionnaires were not returned, while online group dissemination did not allow individual-level verification of exposure to the invitation. Consequently, an accurate overall response rate could not be calculated. A total of 379 questionnaires were returned, exceeding the minimum sample size of 348 indicated by the a priori power analysis. Following data-quality screening, 21 questionnaires were excluded, including eight with incomplete responses, nine showing straight-line response patterns, and four failing the attention-check items. The final analytical sample therefore comprised 358 valid questionnaires, including 109 male participants (30.45%) and 249 female participants (69.55%), corresponding to a valid-questionnaire rate of 94.46%. The study was conducted in accordance with the Declaration of Helsinki and approved by the Ethics Committee of Changsha Normal University.

### 3.2 Measures

All psychometric instruments selected for this study are well-established scales with documented reliability and validity in international and Chinese academic contexts. Responses to the core psychological constructs were captured using Likert-type scaling. To ensure structural consistency across latent constructs and to mitigate potential response biases arising from shifting cognitive frames among rural respondents, all instruments were standardized to a 7-point Likert-type response format. Methodological research indicates that 7-point scales facilitate optimal variance and reliability in self-report psychometrics, particularly within non-academic populations, without compromising the inherent psychometric integrity of the original items (Revilla et al., 2014).

#### 3.2.1 Community embedded support (CES)

Perceived community embedded support was assessed using an adapted version of the Perceived Community Support Questionnaire (PCSQ) originally developed by Herrero and Gracia (2007). To capture the unique socio-cultural realities of contemporary rural China, four core items measuring community integration and participation were extracted from the original scale (e.g., “I feel that I am truly a part of this rural community”). To complement these, four localized items were developed specifically to evaluate both formal institutional and informal communal family education support available within the rural revitalization framework (e.g., “The village committee or local community regularly organizes lectures or mutual-aid activities concerning family education”). The resulting 8-item composite scale utilized a 7-point Likert response format ranging from 1 (strongly disagree) to 7 (strongly agree). Higher aggregate scores indicated stronger perceived community embedded support. The inclusion of these four localized items was informed by a rigorous content validity process, wherein the items were reviewed by a panel of experts in educational sociology and rural psychology to ensure conceptual alignment with the rural revitalization framework and semantic clarity for rural participants. The combined 8-item scale displayed high internal consistency (α = .922) and strong factor loadings, confirming its empirical applicability to our rural sample.

#### 3.2.2 Psychological resilience (PR)

Psychological resilience was measured using an adapted 8-item version of the Connor-Davidson Resilience Scale (CD-RISC-10), originally refined by Campbell-Sills and Stein (2007) and psychometrically validated in the Chinese context by Wang L. et al. (2010). This uni-dimensional instrument captures an individual’s dynamic psychological capacity to adapt positively to adversity. During the initial pilot phase, item-total correlation and factor loading analyses revealed that two items (concerning physiological recovery and humorous coping) exhibited inadequate discriminative power and poor factor loadings within the specific context of rural child-rearing. Consequently, these were excluded to improve the internal consistency and structural validity of the latent construct, resulting in a more robust 8-item instrument that demonstrated superior psychometric properties in our rural sample. Items were rated on a 7-point frequency scale ranging from 1 (never) to 7 (almost always), where higher aggregate scores reflected greater internal psychological resilience.

#### 3.2.3 Parenting stress (PS)

Parenting stress was operationalized using the Parental Distress (PD) subscale from the Parenting Stress Index-Short Form (PSI-SF; Abidin, 1995). The PD subscale comprises 12 items assessing distress associated with parenting responsibilities, perceived parenting competence, and restrictions on personal freedom (e.g., “Since having this child, I feel that I am trapped and unable to do the things I want to do”). Responses were scored on a 7-point Likert scale ranging from 1 (strongly disagree) to 7 (strongly agree). Higher cumulative scores indicated greater self-reported parenting distress.

#### 3.2.4 Subjective well-being (SWB)

Subjective well-being was evaluated using the 5-item World Health Organization Well-Being Index (WHO-5; Topp et al., 2015). The WHO-5 is a globally recognized instrument designed to assess the positive affective components of emotional well-being over the preceding two weeks. It consists of five positively phrased items (e.g., “I have felt cheerful and in good spirits”). Respondents rated items on a 7-point scale from 1 (at no time) to 7 (all of the time). The brevity of this tool minimized cognitive fatigue, making it highly appropriate for rural populations.

### 3.3 Data analysis strategy

Statistical analyses were executed sequentially utilizing SPSS 26.0 and AMOS 26.0. First, descriptive statistics and Pearson bivariate correlation analyses were conducted in SPSS to examine the preliminary relationships among the study variables. Given that all constructs were measured via self-report surveys, potential threats from Common Method Bias (CMB) were systematically monitored. In addition to Harman’s single-factor test in SPSS, a confirmatory factor analysis (CFA) approach was utilized in AMOS to compare the model fit of a single-factor model against the hypothesized measurement model, to assess whether a single common factor was likely to account for the observed covariance among the measures.

Second, a measurement model was specified in AMOS 26.0 using confirmatory factor analysis (CFA) to verify the psychometric properties of the latent constructs. Convergent validity was established using the criteria of average variance extracted (AVE) exceeding 0.50 and composite reliability (CR) exceeding 0.70. Discriminant validity was confirmed using the Fornell and Larcker criterion, ensuring the square root of the AVE for each construct was greater than its inter-construct correlations. Goodness-of-fit was evaluated against standard empirical criteria: χ^2^*/df* < 3, Comparative Fit Index (CFI) > 0.90, Tucker-Lewis Index (TLI) > 0.90, and Root Mean Square Error of Approximation (RMSEA) < 0.08.

Third, the hypothesized integrative moderated mediation model was tested entirely within the structural equation modeling (SEM) framework using AMOS 26.0. To assess the mediation pathway (CES → PR → SWB), a bias-corrected percentile Bootstrap procedure with 5,000 resamples was implemented, where indirect effects were considered statistically significant if the 95% confidence interval (CI) excluded zero. To test the moderation effect of parenting stress, a latent interaction approach was employed (Marsh et al., 2004). Specifically, the unconstrained product-indicator method was utilized. To prevent severe multicollinearity, the observed indicators for both psychological resilience and parenting stress were first mean-centered. Subsequently, matched product terms were generated by multiplying the mean-centered indicators to form the new latent interaction construct (PR × PS). This latent interaction term, alongside the main effects, was simultaneously entered into the structural model to predict SWB. Finally, a simple slope analysis was plotted to visualize the differential impact of psychological resilience on SWB at varying levels of parenting stress ( ± 1 standard deviation from the mean).

## 4 Results

### 4.1 Measurement model: reliability and validity

To rigorously assess potential Common Method Bias (CMB), we employed both exploratory and confirmatory approaches. First, Harman’s single-factor test revealed that the first unrotated factor accounted for 39.41% of the total variance, which is below the conventional 40% threshold. To further validate this finding, a CFA single-factor model comparison was conducted in AMOS. The results demonstrated that the fit of the single-factor model (χ^2^/*df* = 8.629, RMSEA = 0.146, CFI = 0.542, GFI = 0.356, TLI = 0.511) was substantially worse than that of the hypothesized theoretical model (χ^2^*/df* = 1.480, RMSEA = 0.037, CFI = 0.979, GFI = 0.912, TLI = 0.976). Together, these results suggested that a single common factor was unlikely to account for the overall pattern of covariance among the measures.

Before testing the structural relationships, a Confirmatory Factor Analysis (CFA) was conducted to evaluate the reliability and convergent validity of the measurement model. As shown in Table 1, all constructs demonstrated excellent internal consistency. The Cronbach’s α values for Community Embedded Support (CES), Psychological Resilience (PR), Parenting Stress (PS) and Subjective Well-Being (SWB) ranged from 0.892 to 0.947, all well above the recommended threshold of 0.80. Additionally, the Composite Reliability (CR) values ranged from 0.893 to 0.948, exceeding the strict criterion of 0.80 and indicating high construct reliability.

**TABLE 1:** Measurement model: reliability and convergent validity.

Constructs/Item	Unstd.	SE	*T*-value	*P*	Std.	SMC	CR	AVE	Cronbach’s alpha
*Community Embedded Support (CES)* (Herrero and Gracia, 2007)							0.923	0.599	0.922
I feel that I am truly a part of this rural community	1.000				0.723	0.523			
I identify closely with my village and feel a strong sense of belonging among my neighbors	0.983	0.066	14.869	***	0.804	0.646			
I regularly take part in social activities and community events organized in my village	1.031	0.070	14.777	***	0.799	0.638			
I actively respond to calls for support and collaborate with others in local community initiatives	0.998	0.068	14.743	***	0.797	0.635			
The village committee or local community regularly organizes lectures or mutual-aid activities concerning family education	1.041	0.069	15.125	***	0.817	0.667			
Local rural institutions (e.g., village councils, rural schools) provide accessible resources or guidance to help families navigate child-rearing challenges	0.957	0.071	13.444	***	0.729	0.531			
When facing difficulties in educating my children, I can easily turn to experienced neighbors or fellow villagers for advice and informal support	0.964	0.069	13.981	***	0.757	0.573			
There is a strong culture of mutual assistance among families in this village regarding child supervision and sharing educational experiences	1.004	0.071	14.084	***	0.762	0.581			
*Psychological Resilience (PR)* (Campbell-Sills and Stein, 2007; Wang L. et al., 2010)							0.941	0.665	0.941
I am able to adapt when changes occur	1.000	0.851	0.724						
I can deal with whatever comes my way	0.985	0.051	19.347	***	0.817	0.667			
Having to cope with stress can make me stronger	0.937	0.048	19.663	***	0.825	0.681			
I believe I can achieve my goals, even if there are obstacles	0.992	0.050	19.880	***	0.831	0.691			
Under pressure, I stay focused and think clearly	0.974	0.051	19.130	***	0.812	0.659			
I am not easily discouraged by failure	0.922	0.050	18.269	***	0.789	0.623			
I think of myself as a strong person when dealing with life’s challenges	0.949	0.051	18.792	***	0.803	0.645			
I am able to handle unpleasant or painful feelings like sadness, fear, and anger	0.973	0.053	18.512	***	0.795	0.632			
*Parenting Stress (PS)* (Abidin, 1995)							0.948	0.603	0.947
I feel trapped by my responsibilities as a parent	1.000	0.776	0.602						
Since having this child, I have been unable to do new and different things	0.897	0.061	14.756	***	0.729	0.531			
I feel that since having this child, I have been unable to do the things I want to do	0.870	0.052	16.697	***	0.806	0.650			
I feel that I am not the person I used to be—I feel confined by my responsibilities as a parent	0.874	0.057	15.322	***	0.752	0.566			
I feel that I am overwhelmed by the responsibilities of being a parent	1.016	0.064	15.853	***	0.773	0.598			
I feel that I am not able to handle the stress of being a parent as well as I could before	0.990	0.064	15.513	***	0.760	0.578			
I have had many problems coping with the responsibilities of being a parent	1.106	0.066	16.735	***	0.807	0.651			
I feel that I have lost some of my personal freedom since having this child	1.001	0.064	15.609	***	0.763	0.582			
I find it hard to be a parent, and I am often frustrated	0.999	0.061	16.490	***	0.798	0.637			
I feel that my child’s needs are more important than my own	1.028	0.066	15.547	***	0.761	0.579			
I often feel that I am not doing a good job as a parent	0.964	0.061	15.849	***	0.773	0.598			
I feel that I am not receiving enough support in my role as a parent	0.951	0.056	16.873	***	0.813	0.661			
*Subjective Well-Being (SWB)* (Topp et al., 2015)							0.893	0.625	0.892
I have felt cheerful and in good spirits	1.000	0.748	0.560						
I have felt calm and relaxed	1.033	0.071	14.522	***	0.777	0.604			
I have felt active and vigorous	0.980	0.065	15.014	***	0.802	0.643			
I woke up feeling fresh and rested	1.057	0.069	15.342	***	0.819	0.671			
My daily life has been filled with things that interest me	1.041	0.069	15.092	***	0.806	0.650			

SE, Standard Error; *P*, *p*-value; SMC, Squared Multiple Correlations; CR, Composition reliability; AVE, Average variance extracted. ***p < 0.001.

Convergent validity was strongly supported by multiple empirical indicators. First, the Average Variance Extracted (AVE) values for all constructs ranged from 0.599 to 0.665, successfully surpassing the acceptable level of 0.50. Furthermore, all standardized factor loadings were statistically significant (*p* < 0.001) and ranged from 0.723 to 0.851, which is well above the acceptable 0.60 threshold. The Squared Multiple Correlation (SMC) values ranged from 0.523 to 0.724, confirming that the items shared a substantial amount of variance with their respective latent constructs.

Discriminant validity was assessed using the rigorous (Fornell and Larcker, 1981) criterion. As presented in Table 2, the square root of the AVE for each construct (shown in bold on the diagonal, ranging from 0.774 to 0.815) was strictly greater than its highest correlation with any other latent construct. For instance, the highest inter-construct correlation observed was between Psychological Resilience and Subjective Well-Being (*r* = 0.737), which remained well below their respective AVE square roots (0.815 and 0.791). This confirmed that the constructs in the model are empirically distinct. Additionally, Table 2 presents the descriptive statistics (means and standard deviations) and bivariate correlation coefficients among all latent variables, providing an intuitive overview of their initial interrelationships.

**TABLE 2:** Discriminant validity (Fornell and Larcker, 1981).

Constructs	Mean	SD	PS	SWB	PR	CES
PS	4.258	1.069	**0.777**	**0.791**	**0.815**	**0.774**
SWB	4.560	1.195	−0.481			
PR	4.664	1.134	−0.374	0.737		
CES	3.984	1.527	−0.288	0.603	0.599	

Bold values on the diagonal represent the square root of the AVE. Off-diagonal values are the correlations between constructs.

### 4.2 Structural model and hypothesis testing

Before evaluating the structural pathways and testing the hypothesized relationships, the overall model fit of both the baseline mediation model and the final moderated mediation model was thoroughly assessed. As compiled in Table 3, the baseline mediation model exhibited a good fit to the empirical data (χ^2^ = 312.813, *df* = 186, χ^2^*/df* = 1.682, GFI = 0.921, AGFI = 0.902, CFI = 0.976, TLI = 0.970, RMSEA = 0.044, SRMR = 0.036). Crucially, the final hypothesized moderated mediation model demonstrated an even more robust and excellent fit across multiple indices: χ^2^ = 467.770, *df* = 316, χ^2^*/df* = 1.480, CFI = 0.979, TLI = 0.976, RMSEA = 0.037, and SRMR = 0.056. Although the Adjusted Goodness of Fit Index (AGFI = 0.895) fell marginally below the conventional 0.90 threshold, this minor deviation is acceptable given the high complexity of a structural model featuring latent interaction terms, as AGFI is known to be strictly sensitive to model parameters and sample size. Following the two-index strategy and joint criteria recommended by Hu and Bentler (1999), the convergence of excellent CFI, TLI, and RMSEA values provided compelling evidence that the structural model fits the empirical data exceptionally well.

**TABLE 3:** Model fit indices.

Fit indices	χ ^2^	DF	χ ^2^/DF	GFI	RMSEA	CFI	NFI	AGFI	SRMR
Reference value	–	–	< 3	> 0.9	< 0.08	> 0.9	>0.9	> 0.9	< 0.08
Mediation model	312.813	186	1.682	0.921	0.044	0.976	0.943	0.902	0.036
Moderated mediation model	467.770	316	1.480	0.912	0.037	0.979	0.938	0.895	0.056

χ^2^, Chi-Square Minimum Fit Function; DF, Degrees of Freedom; χ^2^/DF, Chi-Square to Degrees of Freedom ratio; GFI, Goodness of Fit Index; AGFI, Adjusted Goodness of Fit Index; CFI, Comparative Fit Index; RMSEA, Root Mean Square Error of Approximation; NFI, Normed Fit Index; SRMR, Standardized Root Mean Square Residual.

To isolate the unique effects of the core theoretical constructs, all structural paths were estimated while concurrently controlling for demographic variables (including parents’ age and gender). Statistical analysis indicated that these control variables did not exert any significant effects on either Psychological Resilience (PR) or Subjective Well-Being (SWB), thereby ensuring that the observed variances were uniquely explained by our focal predictors.

The standardized and unstandardized path coefficients for the structural model are summarized in Table 4. In alignment with Hypothesis 1 (H1), Community Embedded Support (CES) was found to be a significant positive predictor of Subjective Well-Being (SWB) (*B* = 0.157, *S.E.* = 0.043, *C.R.* = 3.635, *p* < 0.001, β = 0.198). This confirms that higher levels of external social capital and structural support within rural communities are strongly associated with elevated psychological well-being of parents, supporting H1.

**TABLE 4:** Summary of structural path coefficients and hypothesis testing results.

Hypotheses	Construct	Unstandardized path coefficient	S.E.	C.R.	*P*	Standardized path coefficient	Results
H1	CES→SWB	0.157	0.043	3.635	***	0.198	Supported
H2a	CES→PR	0.492	0.048	10.187	***	0.604	Supported
H2b	PR→SWB	0.544	0.057	9.490	***	0.559	Supported
H3	PRxPS→SWB	−0.173	0.044	−3.960	***	−0.161	Supported

***p < 0.001. All variables were mean centered before the creation of the latent interaction term (PRxPS) to reduce multicollinearity.

Regarding the component paths underlying the proposed mediation mechanism, CES was significantly and positively associated with Psychological Resilience (PR) (*B* = 0.492, SE = 0.048, C.R. = 10.187, *p* < 0.001, β = 0.604), supporting H2a. In turn, Psychological Resilience was significantly and positively associated with SWB (*B* = 0.544, SE = 0.057, C.R. = 9.490, *p* < 0.001, β = 0.559), supporting H2b. Together, these findings provide the necessary path-level evidence for the proposed mediation mechanism. The overall indirect effect specified in H2 was subsequently evaluated using the bootstrap mediation analysis reported in section 4.3.

Most critically, to examine the boundary conditions governing this resource translation process (Hypothesis 3), the latent interaction term (PR × PS) was introduced into the structural model. The analysis revealed that the interaction between Psychological Resilience and Parenting Stress exerted a significant negative effect on SWB (*B* = −0.173, *S.E.* = 0.044, *C.R.* = −3.960, *p* < 0.001, β = −0.161). The negative sign of this interaction coefficient confirms a strong buffering or attenuation effect, indicating that the relationship between psychological resilience and subjective well-being is not static but changes significantly as a function of chronic caregiving stress. Therefore, Hypothesis 3 is fully supported.

### 4.3 Mediation analysis

To evaluate the mediating role of Psychological Resilience (PR) in the relationship between Community Embedded Support (CES) and Subjective Well-Being (SWB), a bias-corrected percentile Bootstrap procedure with 5,000 resamples was conducted. As delineated in Table 5, the total effect of CES on SWB was positive and statistically significant (*B* = 0.425, SE = 0.053, *Z* = 8.019, 95% CI [0.329, 0.539]).

**TABLE 5:** Mediation analysis.

SIE	Point estimate	Product of coefficients		Bootstrapping = 5,000				Ratio %
				Bias-corrected 95%CI		Percentile 95%CI		
		SE	*Z*	Lower	Upper	Lower	Upper	
Indirect effect								
CES-PR-SWB	0.268	0.044	6.091	0.192	0.366	0.188	0.363	63.06%
Direct effect								
CES-SWB	0.157	0.044	3.568	0.071	0.245	0.071	0.245	36.94%
Total effect								
CES-SWB	0.425	0.053	8.019	0.329	0.539	0.327	0.536	100%
Model without mediators								
CES-SWB	0.477	0.058	8.224	0.371	0.600	0.370	0.599	–

Unstandardized estimating of 5,000 bootstrap sample. All reported estimates for indirect, direct, and total effects are unstandardized coefficients. The ratio % (percentage mediated) was calculated by dividing the unstandardized indirect effect by the unstandardized total effect.

Upon the inclusion of Psychological Resilience into the model as a mediator, the direct effect of CES on SWB remained significant (*B* = 0.157, SE = 0.044, *Z* = 3.568, 95% CI [0.071, 0.245]). Crucially, the indirect effect of CES on SWB through PR was highly significant (*B* = 0.268, SE = 0.044, *Z* = 6.091). The robustness of this mediating pathway was rigorously validated, as both the bias-corrected 95% confidence interval [0.192, 0.366] and the percentile 95% confidence interval [0.188, 0.363] strictly excluded zero.

Furthermore, effect size calculations revealed that this internal psychological mechanism holds considerable explanatory power. The indirect pathway (CES → PR → SWB) accounted for a substantial 63.06% of the total effect, while the direct effect accounted for the remaining 36.94%. These empirical findings confirm that psychological resilience serves as a significant partial mediator in linking external structural support into internal well-being, thereby providing substantial empirical support for Hypothesis 2 (H2).

### 4.4 Moderated mediation analysis

To delineate the boundary conditions of the mediating process and test Hypothesis 3 (H3), the moderating effect of Parenting Stress (PS) on the relationship between Psychological Resilience (PR) and Subjective Well-Being (SWB) was examined. As previously detailed in Table 4, the latent interaction term (PR × PS) exerted a statistically significant negative effect on SWB (*B* = −0.173, *SE* = 0.044, *C.R.* = −3.960, *p* < 0.001, β = −0.161). The negative coefficient of this interaction term indicates that parenting stress acts as a significant moderator, specifically attenuating the link between psychological resilience and subjective well-being.

To formally test the proposed second-stage moderated mediation, the index of moderated mediation and the conditional indirect effects were estimated using 5,000 bias-corrected bootstrap samples. As shown in Table 6, the index of moderated mediation was negative and statistically significant (index = −0.085, SE = 0.025, 95% bias-corrected CI [−0.143, −0.047], *p* < 0.001), as the confidence interval did not contain zero. This finding indicates that the indirect association between community embedded support and subjective well-being through psychological resilience became weaker as parenting stress increased. Specifically, the conditional indirect association was strongest at low parenting stress (indirect effect = 0.359, SE = 0.061, 95% bias-corrected CI [0.254, 0.496], *p* < 0.001), decreased at the mean level of parenting stress (indirect effect = 0.268, SE = 0.044, 95% bias-corrected CI [0.192, 0.367], *p* < 0.001), and was weakest at high parenting stress (indirect effect = 0.177, SE = 0.040, 95% bias-corrected CI [0.103, 0.263], *p* < 0.001). Although the conditional indirect association remained statistically significant at all three levels, its magnitude was approximately 50.7% smaller under high than under low parenting stress. These results provide formal support for the proposed second-stage moderated mediation model and Hypothesis 3.

**TABLE 6:** Conditional indirect effects and index of moderated mediation.

Effect	Level of parenting stress	Estimate	SE	Lower 95% CI	Upper 95% CI	*p*
Conditional indirect effect	Low (−1 SD)	0.359	0.061	0.254	0.496	<0.001
Conditional indirect effect	Mean	0.268	0.044	0.192	0.367	<0.001
Conditional indirect effect	High (+1 SD)	0.177	0.040	0.103	0.263	<0.001
Index of moderated mediation	–	−0.085	0.025	−0.143	−0.047	<0.001

Estimates are unstandardized. Confidence intervals were calculated using 5,000 bias-corrected bootstrap samples. Parenting stress was mean-centered; low and high levels represent one standard deviation below and above the mean, respectively. SE, bootstrap standard error; CI, confidence interval.

**FIGURE 2:**
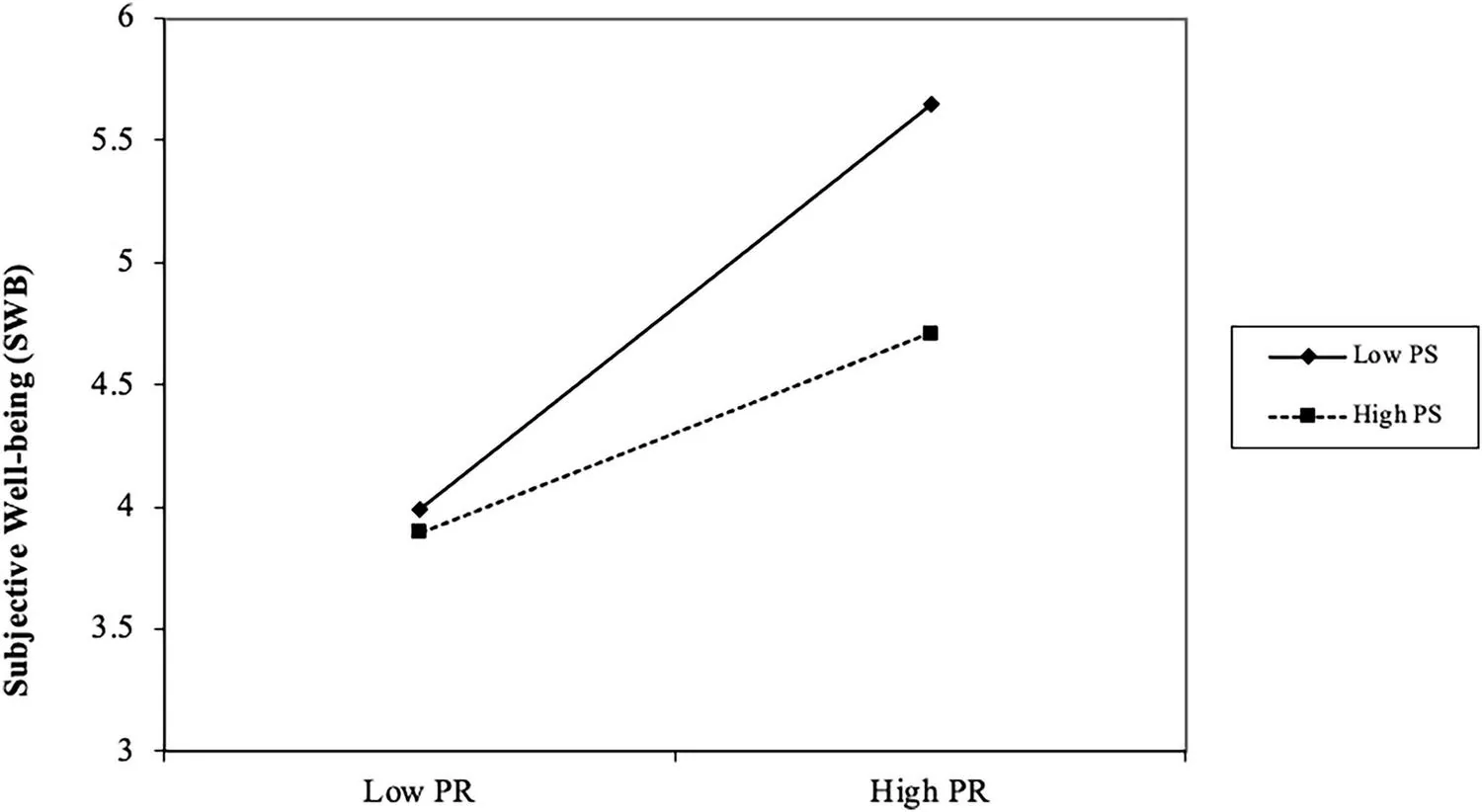
Slope chart.

To further unpack the nature of this interaction and visually validate the structural moderation, a simple slope analysis was plotted (Figure 2). The graph delineates the trajectory of the relationship between psychological resilience and SWB at conditional levels of parenting stress (operationalized as Low PS and High PS). The simple slope chart clearly demonstrates a divergent pattern: for parents experiencing low levels of parenting stress (illustrated by the solid line), psychological resilience maintains a robust and steep positive association with subjective well-being. Conversely, for parents enduring high levels of parenting stress (illustrated by the dashed line), this positive association is significantly attenuated, resulting in a noticeably flatter slope.

These findings align with the resource depletion mechanism posited in our theoretical framework. They empirically indicate that when rural parents are confronted with chronic, high-intensity parenting stress, the protective efficacy of their psychological resilience is substantially attenuated. Under such demanding conditions, it is plausible that the accumulated psychological resilience is disproportionately consumed merely to cope with immediate caregiving stressors, leaving insufficient residual psychological capacity to support overall subjective well-being. Taken together, the significant interaction, conditional indirect effects, and index of moderated mediation provide convergent evidence that parenting stress attenuates the latter stage of the indirect pathway from community embedded support to subjective well-being through psychological resilience. Hypothesis 3 is therefore fully supported.

## 5 Discussion

### 5.1 Summary of main findings

Set against the backdrop of China’s rural revitalization and the pervasive demands of intensive parenting, this study sought to delineate the mechanisms linking external ecological resources to internal parental well-being. By empirically testing an integrative moderated mediation framework, the current research yielded several critical findings. First, consistent with our expectations, community embedded support (CES) was positively associated with the subjective well-being (SWB) of rural parents. Second, psychological resilience emerged as a significant partial mediator, indicating that external community resources are systematically linked to enhanced well-being through the facilitation of internal psychological adaptability. Finally, the significant index of moderated mediation confirmed that parenting stress conditioned the indirect association between community embedded support and subjective well-being through psychological resilience. Although the conditional indirect association remained significant across all examined levels of parenting stress, its magnitude decreased from 0.359 at low parenting stress to 0.177 at high parenting stress. Thus, psychological resilience remained relevant to parental well-being under high stress, but its role in translating community support into well-being was substantially attenuated.

### 5.2 Theoretical implications

The present findings refine the application of demand–resource logic to family well-being by integrating the Family Demands-Resources perspective, Conservation of Resources theory, and ecological approaches. The findings extend demand–resource perspectives by showing that community support, psychological resilience, and parenting stress are jointly associated with rural parents’ SWB. In particular, the results connect a meso-level community resource with an individual psychological resource and identify parenting stress as a condition associated with variation in the indirect association.

First, community embedded support was positively associated with the subjective well-being of rural parents. This finding is consistent with evidence that social support is positively related to subjective well-being among Chinese parents of children with autism spectrum disorder (Ban et al., 2021) and that the effectiveness and perceived availability of support are associated with higher subjective well-being among Chinese mothers of children with autism spectrum disorder (Bi et al., 2022). It also converges with Wu et al. (2022), who found that agricultural cooperative membership was associated with the subjective well-being of rural Chinese households partly through income and social capital. However, Tang et al. (2021) reported no significant difference in subjective well-being between participants and non-participants in a targeted poverty-alleviation program in rural China. One possible explanation for this difference concerns the nature of the resources examined. Participation in a top-down program or access to material opportunities may not necessarily provide the routinely available relationships, sense of belonging, trusted guidance, and reciprocal assistance captured by community embedded support. Consistent with the concept of collective efficacy (Sampson et al., 1997), the present finding therefore highlights the potential relevance of accessible and relationally embedded community resources for rural parents.

Second, psychological resilience partially mediated the association between community embedded support and subjective well-being. The indirect association was 0.268 and accounted for 63.06% of the total association, while the direct association remained significant. This result is consistent with Flores-Buils and Andrés-Roqueta (2022), who found that professional support and participation in family associations were associated with parental resilience among parents of children with neurodevelopmental disorders. It also parallels Zhao et al. (2023), whose study of parents of primary-school students in central China showed that perceived family support was positively associated with psychological resilience and that resilience partially mediated the association between family support and parental burnout. Together with evidence linking resilience to subjective well-being in adults (Yıldırım and Arslan, 2022), these findings support the conceptualization of resilience as a contextually facilitated personal resource rather than solely a fixed individual trait (Luthar et al., 2000). The current study extends this evidence from predominantly clinical, disability-related, or urban parenting samples to parents in rural communities and from negative outcomes such as burnout to positive subjective well-being. At the same time, the remaining direct association indicates that community support is not related to well-being exclusively through resilience. Immediate instrumental assistance, social belonging, and access to parenting information may represent additional pathways, although these mechanisms were not directly tested in the present study.

Third, parenting stress constituted a significant boundary condition in the second stage of the indirect association. The index of moderated mediation was −0.085, with a 95% bias-corrected confidence interval of [−0.143, −0.047]. Moreover, the conditional indirect association decreased from 0.359 at low parenting stress to 0.177 at high parenting stress, representing an attenuation of approximately 50.7%. This pattern complements Zhao et al. (2023), who found that resilience weakened the positive association between parenting stress and parental burnout. Whereas their analysis conceptualized resilience as a resource that buffers the adverse consequences of stress, the present model examines the complementary side of the demand–resource interaction: increasing parenting stress restricts the extent to which resilience is associated with positive well-being. This result is also compatible with the resource-imbalance perspective underlying research on parental burnout across countries (Roskam et al., 2021) and with family stress frameworks suggesting that persistent demands consume resources required for adaptive functioning (Belsky, 1984; Conger and Conger, 2002). Importantly, however, the conditional indirect association remained statistically significant even at high parenting stress. The findings therefore do not indicate that resilience becomes ineffective; rather, its role in translating community support into well-being is substantially attenuated. This pattern is consistent with a resource-depletion explanation.

#### 5.3 Practical implications

The findings have implications for parents, families, rural communities, practitioners, and policymakers. First, village committees and rural schools should translate community embedded support into predictable and routinely accessible services rather than relying primarily on occasional activities. Possible measures include parent mutual-aid groups, scheduled family-education consultation sessions, community childcare or respite arrangements, and clearly communicated referral pathways for educational, psychological, and social services. Because psychological resilience only partially mediated the association between community support and well-being, these initiatives may assist parents both by providing immediate practical and emotional resources and by creating supportive contexts in which adaptive coping capacities can be strengthened.

Second, practitioners should avoid treating resilience training as a sufficient stand-alone response to severe parenting stress. Community workers, school counselors, and family-education practitioners could use brief and non-stigmatizing screening procedures to identify parents experiencing elevated stress. Parents with low or moderate stress may benefit from group-based coping, emotion-regulation, and resilience-building programs. For parents experiencing high stress, however, such programs should be combined with measures that reduce actual parenting demands, including accessible childcare, respite services, professional parenting guidance, co-parenting support, and referral to psychological or financial assistance when necessary. Because the indirect association remained significant at high parenting stress, resilience-oriented support should not be abandoned; instead, it should be delivered together with structural demand-reduction strategies.

Third, rural family policy should prioritize stable support infrastructure rather than one-off informational or economic initiatives. Local governments could provide sustained funding for village-level family services, train village workers and rural school personnel to identify and refer highly stressed parents, and ensure that services remain accessible to parents with limited mobility or digital access. Within families, a more equitable division of caregiving responsibilities between partners and appropriate intergenerational assistance may also help reduce the concentration of parenting demands on a single caregiver. Future community programs should monitor service uptake, parenting stress, resilience, and subjective well-being over time so that their effectiveness can be evaluated rather than assumed.

### 5.4 Limitations and future directions

Despite its robust theoretical grounding, this study has several limitations that warrant consideration. First, the cross-sectional design precludes the establishment of definitive causal directions among community support, resilience, parenting stress, and subjective well-being. While our integrative model is anchored in established theoretical frameworks, future research should employ longitudinal cross-lagged panel models or Ecological Momentary Assessment (EMA) to capture the dynamic, day-to-day fluctuations of resource accumulation and depletion. Second, the reliance on self-report measures introduces the potential for common method bias (CMB). Although rigorous statistical monitoring indicated that CMB was not a pervasive issue in this study, future investigations could benefit from incorporating multi-informant approaches (e.g., spouse or teacher evaluations of parenting stress). Third, the multi-site convenience sampling strategy and mixed-mode recruitment procedure introduce potential self-selection and non-response bias. Because online invitations were disseminated through village collective WeChat groups and some paper questionnaires were not returned, the total number of eligible parents who received or viewed the invitation could not be determined, preventing the calculation of an accurate overall response rate. Future studies should use household registries or clearly documented sampling frames, record invitations and returns separately for each recruitment mode, and, where feasible, adopt stratified probability sampling to improve representativeness. Finally, the sample was exclusively drawn from rural parents in China. The unique socio-cultural dynamics of Chinese rural revitalization and intensive parenting norms may limit the cross-cultural generalizability of the findings. Future research should aim for cross-cultural validations and comparisons with urban populations to further test the universality of this conditional indirect effect model.

## 6 Conclusion

This study highlights the interplay among ecological resources, internal psychological capacities, and chronic caregiving demands in shaping the subjective well-being of rural parents. Community embedded support was positively associated with subjective well-being both directly and indirectly through psychological resilience, while parenting stress significantly attenuated the latter stage of this indirect association. Because the conditional indirect association remained significant even at high parenting stress, resilience should be understood as a context-sensitive resource whose contribution is weakened, rather than eliminated, by intensive parenting demands. These findings suggest the value of considering both personal resilience and structural support when developing services for rural parents.

## Data Availability

The original contributions presented in the study are included in the article/Supplementary material, further inquiries can be directed to the corresponding author.
